# Supplementary material for: Tracing the trajectories of SARS-CoV-2 variants of concern between December 2020 and September 2021 in the Canary Islands (Spain)
Source: Front Cell Infect Microbiol. 2022 Sep 9;12:919346. doi: 10.3389/fcimb.2022.919346 (PMC9504278; doi:10.3389/fcimb.2022.919346)
Supplement: Supplementary file 1 [file Image_1.pdf]

A

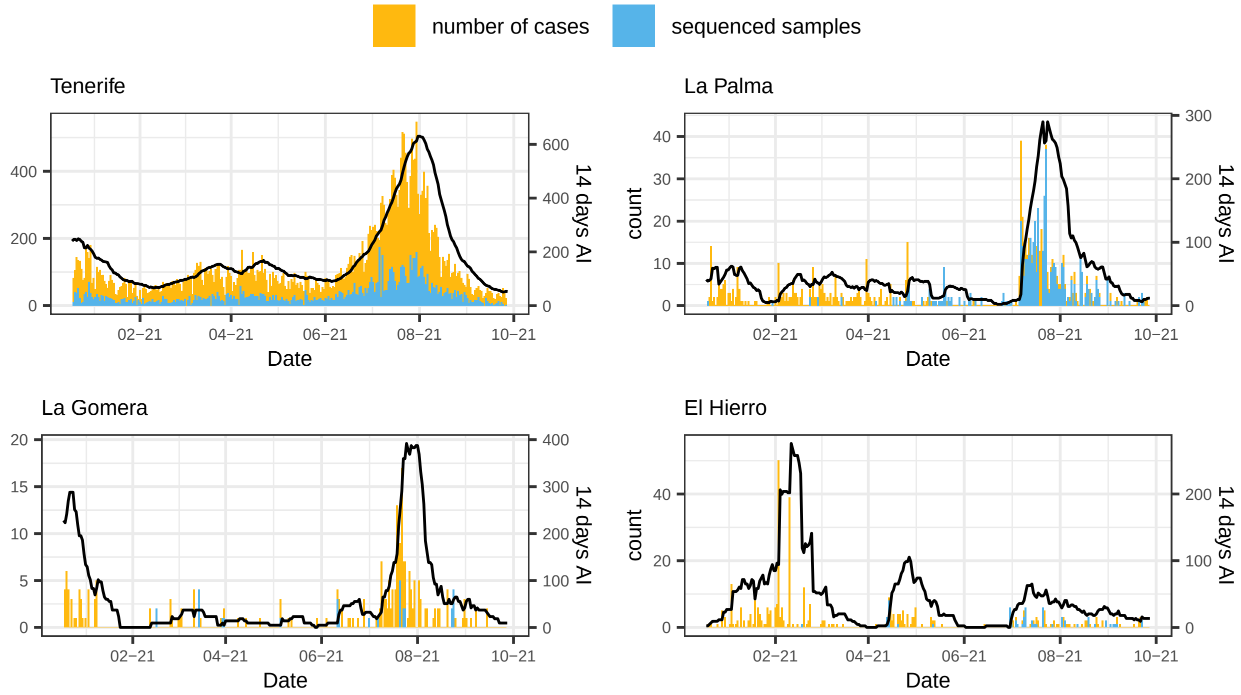
number of cases
sequenced samples
Tenerife
La Palma
La Gomera
El Hierro
count
14 days AI
Date
02−21
04−21
06−21
08−21
10−21

B

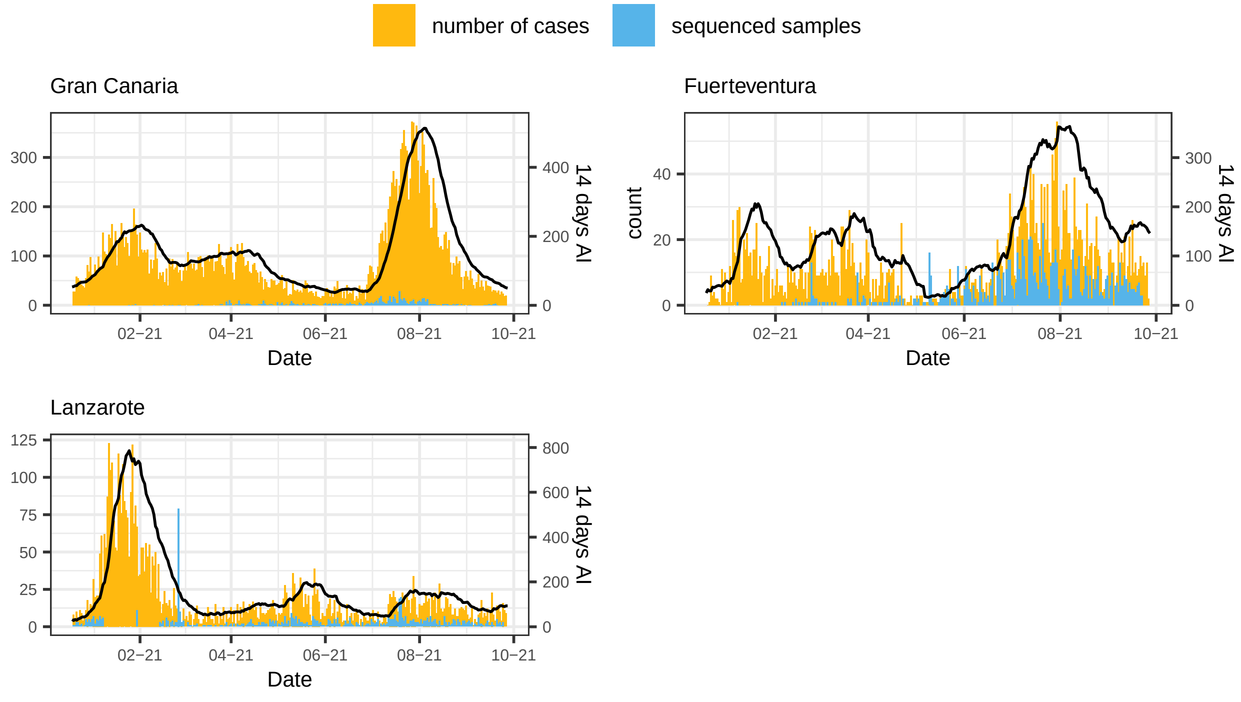
number of cases
sequenced samples
Gran Canaria
Fuerteventura
Lanzarote
count
14 days AI
Date
02−21
04−21
06−21
08−21
10−21
